# Impacts of electrogenerated chemiluminescence mechanism on the emission spectra and intensities

**DOI:** 10.1007/s44211-025-00736-6

**Published:** 2025-02-25

**Authors:** Ryoichi Ishimatsu

**Affiliations:** 1 Department of Applied Physics, University of Fukui, 3-9-1, Bunkyo, Fukui, 910-8507 Japan; 2 Reserach Center for Fibers and Materials, University of Fukui, 3-9-1, Bunkyo, Fukui, 910-8507 Japan

**Keywords:** Electrochemiluminescence, Kinetics, Electron transfer, Redox potentials

## Abstract

In this mini-review, recent progress in electrogenerated chemiluminescence (ECL) reported by our group is mainly introduced. First, the general principles of ECL and standard molecules for ECL are described, and then the ECL properties of thermally activated delayed fluorescence (TADF) molecules, pyrrolopyrrole aza-BODIPYs (PPABs), pyrene (Py) and its derivative, Eu(III) and Tb(III) complexes are introduced. The ECL efficiency of TADF molecules is increased by spin up-conversion from the T_1_ to S_1_ state, and that of PPABs is incremented kinetically due to the slower rate for the T_1_ state formation than the S_1_ state formation. The excimer formation mechanism of Py and its derivative in ECL is discussed. It is shown that very sharp ECL spectra can be obtained from Eu(III) and Tb(III) complexes. Furthermore, intense ECL for binary mixed systems using Ir(ppy)_3_ and electron donor or acceptor molecules is described. These results provide insight into how the ECL mechanism affects emission intensity, efficiency, and spectrum.

**Graphical abstract:**

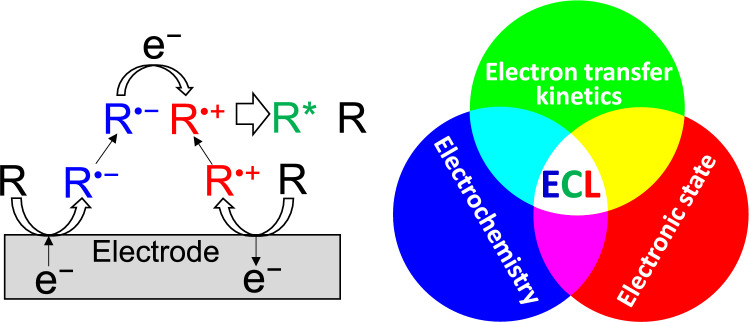

## Introduction

### Principle of ECL

Light emission phenomena induced by radical species generated by electrochemical reactions are called electrogenerated chemiluminescence or electrochemiluminescence (ECL) [1, 2]. ECL can mainly be categorized into annihilation and coreactant systems. The annihilation ECL can be written as,

1 $$\text{R}+\text{e}\rightleftharpoons {\text{R}}^{\bullet -} \left(\text{Electrochemical reduction}\right)$$

2 $$\text{R}-\text{e}\rightleftharpoons {\text{R}}^{\bullet +} \left(\text{Electrochemical oxidation}\right)$$

3 $${\text{R}}^{\bullet -}+{\text{R}}^{\bullet +}\to {\text{R}}^{*}+\text{R }\left(\text{Ion annihilation}\right)$$

4 $${\text{R}}^{*}\to \text{R}+h\nu \left(\text{light emission}\right)$$

**Fig. 1:**
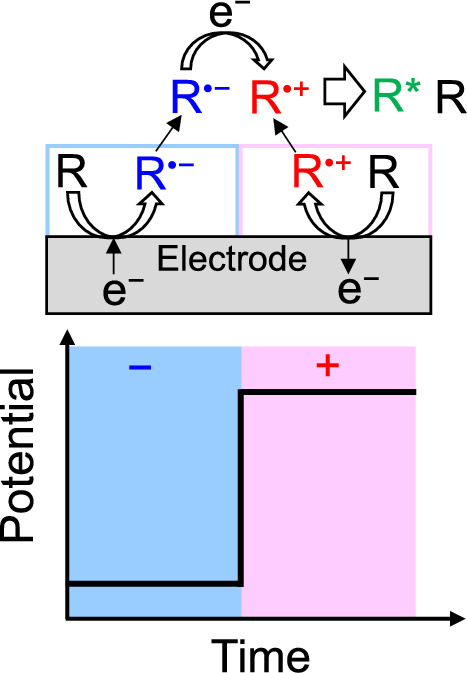
Illustration of annihilation ECL with a potential step method

The generation of ECL with a potential step method is illustrated in Fig. 1. The excited state (R*) of a photoluminescent (PL) molecule (R) can be produced by ion annihilation of the electrochemically generated radical anion (R^•^^−^) and cation (R^•^^+^), and subsequently, the ECL can be seen through the radiative transition of R*.

Single ECL systems are systems in which one kind of light-emitting molecule is used, and mixed ECL systems are systems in which several molecules are involved as sources of radical ions. For example, a mixed ECL system with R and D or A [1], where D and A stand for molecules that can electrochemically produce the radical anion (A^•^^−^) and cation (D^•−^), respectively, can be written as,

5 $$\text{A}+\text{e}\rightleftharpoons {\text{A}}^{\bullet -} \left(\text{Electrochemical reduction}\right)$$

6 $$\text{D}-\text{e}\rightleftharpoons {\text{D}}^{\bullet +} \left(\text{Electrochemical oxidation}\right)$$

7 $${\text{R}}^{\bullet -}+{\text{D}}^{\bullet +}\to {\text{R}}^{*}+\text{ D }\left(\text{Ion annihilation}\right)$$

8 $${\text{R}}^{\bullet +}+{\text{A}}^{\bullet -}\to {\text{R}}^{*}+\text{ A }\left(\text{Ion annihilation}\right)$$

The excited state of D and A (D* and A*, respectively) can also be formed, which depends on the energy level of the pair (R^•−^ and D^•+^ or R^•+^ and A^•−^) and that of D* and A*.

In the case of coreactant systems, coreactants can produce an oxidative or reductive species through electrochemical oxidation or reduction. Tripropylamine (TPA) is one of the frequently used coreactant, and the ECL with TPA can be written as [1, 3],

9 $$\text{TPA}-\text{e}\rightleftharpoons {\text{TPA}}^{\bullet +} \left(\text{Electrochemical oxidation}\right)$$

10 $${\text{TPA}}^{\bullet +}-{\text{H}}^{+}\rightleftharpoons {\text{TPA}}^{{\prime}\bullet } \left(\text{deprotonation}\right)$$

11 $${\text{R}}^{\bullet +}+{\text{TPA}}^{{\prime}\bullet }\rightleftharpoons {\text{R}}^{*}+ {\text{TPA}}^{\prime} \left(\text{Formation of excited state}\right)$$

**Fig. 2:**
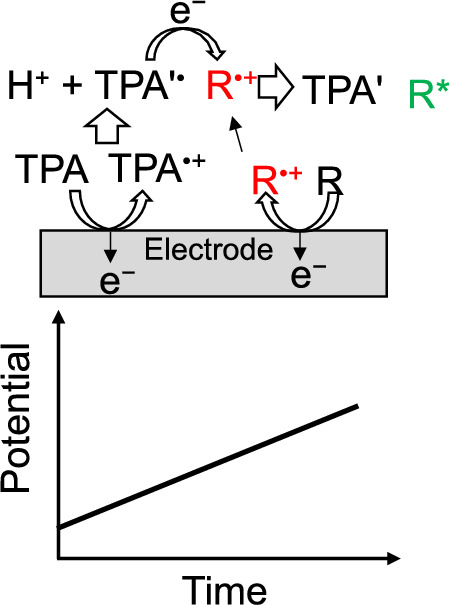
Illustration of coreactant ECL using TPA with a potential sweep method

The generation of ECL using TPA with a potential sweep method is depicted in Fig. 2. The electrochemically oxidized form of TPA (TPA^•+^) turns into a radical product (TPA'^•^) with a negatively large reduction potential (*E*^⚬^(TPA'^•^/TPA') ≈ − 1.7 V vs. SCE [4], where *E*^⚬^ is the standard redox potential) by releasing H^+^. R* will be formed by the electron transfer reaction between R^•+^ and TPA'^•^. Several coreactants such as oxalate ion (C_2_O_4_^2−^, oxidation system, which produces CO_2_^•−^, *E*^⚬^(CO_2_^•−^/CO_2_) =  − 1.9 V vs. NHE [1, 5]), peroxydisulfate (S_2_O_8_^2−^, reduction system, which produces SO_4_^•−^, *E*^⚬^(SO_4_^•−^/SO_4_^2−^) > 3.15 V vs. SCE [6]), benzoyl peroxide (reduction system, which produces C_6_H_5_CO_2_^•^, *E*^⚬^(C_6_H_5_CO_2_^•^/C_6_H_5_CO_2_^−^) > 1.5 V vs. SCE [1, 7]) are known. R* will be formed by the electron transfer reaction between the radical products and R^•−^ or R^•+^. Coreactants are powerful tools to enhance the ECL intensity in the case of weak ECL for single systems due to unstable R^•−^ or R^•+^, or the insufficient energy of a pair of R^•−^ and R^•+^ to produce R*. Coreactants are frequently used for ECL immunoassay [3, 8, 9], which is one of the practical applications of ECL.

## Generation of ECL

For annihilation ECL systems, since R^•−^ and R^•+^ are necessary to collide with each other effectively for the electron transfer reaction to form R*, thin layer cells (with a thickness of ≲10 μm), which are two-electrode systems [10–13], are sometimes employed. The thickness at which ECL can be observed is strongly affected by the stability of the radical ions. For example, with 9,10-diphenylanthracene (DPA) and rubrene, which produce stable radical ions, the ECL could be observed on the whole area of ITO thin-layer electrode (1 × 2 cm) at the thicknesses of 2.6 and 6 μm, respectively. With 1,2,3,5-tetrakis(carbazol-9-yl)-4,6-dicyanobenzene (4CzIPN), whose radical cation is unstable, the ECL was seen using a thin-layer cell with a thickness of 0.9 μm [10]. Although thin-layer ECL cells are practical for fabricating light-emitting devices, the electrode potential is difficult to control well because of an *IR* drop. Thus, for the purpose of analyzing ECL, square wave voltages are frequently applied to the working electrode employing three-electrode systems (Fig. 1). For the coreactant systems, potential sweep (Fig. 2) or constant potential methods are employed.

## Standard substrate

**Fig. 3:**
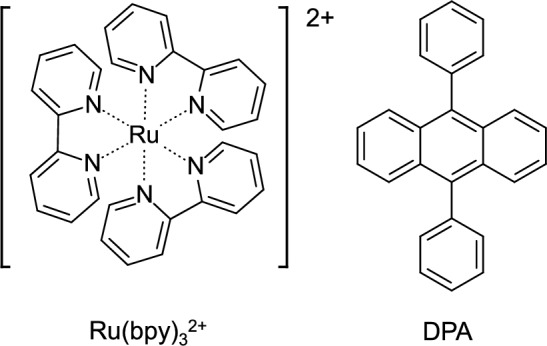
Molecular structure of standard substrates of ECL

The Coulombic ECL efficiency (ϕ_ECL_), which is the value of the total ECL intensity divided by the total charge during the emission of the ECL, is normally determined using a standard substrate. Ru(bpy)_3_^2+^ and DPA (Fig. 3) are frequently used as the standard substrate. ϕ_ECL_ of the target molecule can be obtained from the total charge and total ECL intensity of the target and standard molecules with ϕ_ECL_ of the standard molecules.

The emissive excited state of Ru(bpy)_3_^2+^ (Ru(bpy)_3_^2+^*, the maximum ECL wavelength (λ_ECL_) is about 620 nm) can be produced by the electron transfer reaction between electrochemically generated Ru(bpy)_3_^3+^ and Ru(bpy)_3_^+^. ϕ_ECL_ of Ru(bpy)_3_^2+^ is 5–6% in acetonitrile (MeCN) [14], whereas the photoluminescence quantum yield (PLQY) is about 9% at room temperature [15]. Ru(bpy)_3_^2+^ shows one reversible oxidation wave and three reversible reduction waves, and by the oxidation, Ru(bpy)_3_^3+^, and by the reduction, Ru(bpy)_3_^+^, Ru(bpy)_3_, and Ru(bpy)_3_^−^ can be produced. When the potential is set to generate Ru(bpy)_3_^−^ and Ru(bpy)_3_^3+^, a higher ECL intensity can be seen compared to the case where the potential is set to produce Ru(bpy)_3_^+^ and Ru(bpy)_3_^3+^ [15]. Bard et al. used a rotating ring-disk electrode and changed the reduction potential of the disk, where the potential of the ring was fixed to generate Ru(bpy)_3_^3+^, and found that the ratio of the ECL intensity is about 1: 2: 3 when the reduction potential is enough to produce Ru(bpy)_3_^+^, Ru(bpy)_3_, and Ru(bpy)_3_^−^, respectively [14]. They pointed out that although the ECL intensity changed by the reduction potential, ϕ_ECL_ of Ru(bpy)_3_^2+^ was almost independent of the reduction potential.

DPA, whose PLQY is close to unity, shows blue ECL (λ_ECL_≈ 420 nm). The ECL intensity (and thus, ϕ_ECL_) of DPA significantly depends on the solvent. In the 1970s, ϕ_ECL_ of DPA in *N*,*N*-dimethylformamide was measured with a rotating ring-disk electrode and found to be ϕ_ECL_ < 0.1% [16, 17]. When a mixture solution of MeCN, benzene, and toluene was employed, ϕ_ECL_ of DPA dramatically increased (~ 8%)[18]. Maness and Wightman revealed that ϕ_ECL_ of DPA in a low permittivity solvent, 1,2-dimethoxyethane (its relative permittivity is 7.2) depends on the ionic strength of the supporting electrolyte. They measured steady-state ECL with double-band microelectrode and used Ru(bpy)_3_^2+^ as a standard. The determined ϕ_ECL_ of DPA in 1,2-dimethoxyethane was 9.6 and 23.4% when the concentration of the supporting electrolyte (tetrabutylammonium hexafluorophosphate, TBAPF_6_) was 10^−4^ and 0.1 M, respectively [19]. Itoh et al. reported that ϕ_ECL_ of DPA in MeCN was ~ 2%, which was measured by a potential step method [20]. Wightman et al. determined ϕ_ECL_ of DPA in MeCN to be 5% [21] with a microelectrode where the ECL was generated by high-frequency square-wave voltages (~ 20 kHz, transient ECL) and 6% [22] with a double-band microelectrode (steady state ECL) by using Ru(bpy)_3_^2+^ as a standard. Since the capacitance and *IR* drop can dramatically be suppressed using microelectrodes, such high-frequency generation of the ECL was possible. The solvent-dependent ϕ_ECL_ of DPA could be related to the stability of the radical ions. In polar solvents such as *N*,*N*-dimethylformamide, undesired reactions of the radical ions lower ϕ_ECL_. Such reactions can be suppressed with high-frequency generation of ECL. In the case of using a low permittivity solvent, ion pairing of the radical ions with ions of the supporting electrolyte is responsible for the ionic-strength-dependent ϕ_ECL_ [19]. The ϕ_ECL_ of DPAs is sometimes normalized to unity, and the relative ϕ_ECL_ value of target molecules against the DPA system is reported.

### The common understanding for achieving high ECL efficiency

It is commonly recognized that ECL tends to be highly efficient when (1) R shows high PLQY and (2) produces stable radical cation and anion, and (3) the radical ion pair has sufficient energy to produce the emissive excited state.

(1) R* deactivates to the ground state through the same electronic transitions in the ECL and PL cases, i.e., the rate constant of the radiative and non-radiative transitions from the excited states to the ground state is the same in both cases (it is noted that the non-radiative transitions in both cases may be affected by the concentration of R and supporting electrolyte somewhat). Therefore, R having high PLQYs is favorable for ECL.

(2) When the electrochemically generated radical ions are stable, ion annihilation to form the excited state occurs efficiently.

(3) The gap between the reduction and oxidation potentials that produce R^•−^ and R^•+^, respectively, determines the energy of the pair of R^•−^ and R^•+^ (− *F*Δ*E* = − *F*(*E*_1/2_(R/R^•+^) − *E*_1/2_(R/R^•−^)), where *F* is the faraday constant, and *E*_1/2_(R/R^•+^) and *E*_1/2_(R/R^•−^) stand for the halfwave potential for the generation of the radical cation and anion, respectively). When the energy level of the pair is higher than that of the emissive excited state of R* (S_1_ and T_1_ state for fluorescent and phosphorescent molecules, respectively), the emissive excited state can energetically be formed by ion annihilation.

In case the energy level of a radical pair is insufficient for the formation of the S_1_ state, the T_1_ state will be formed, thus ECL cannot normally seen for fluorescent molecules. However, ECL may be observed through the triplet–triplet annihilation [23–25].

Basic properties including the above properties of R can be revealed from electrochemical and optical measurements. *E*_1/2_ and the radical ion stability can be determined from cyclic voltammograms, and excited-state energy can be obtained from light absorption and fluorescence spectrum measurements. The rate of the radiative transition can be calculated from the results of PLQY and PL lifetime measurements. The theoretical calculations such as density functional theory (DFT) and time-dependent DFT calculations for luminescent molecules can reveal the highest occupied molecular orbital (HOMO) and lowest unoccupied molecular orbital (LUMO), the HOMO–LUMO energy gap, molecular orbitals involved in the electronic transitions due to the light absorption, and energy of the excited states, which are very informative for analyzing the ECL properties.

### Spin statistics in ECL

Radical ions of organic molecules are normally in a doublet state. The spin multiplicity of the radical ion pair is 1 or 3 depending on the combination of the spins of the unpaired electrons of radical ions, so R* generated by the electron transfer is in singlet or triplet states. There are one and three combinations of electron spins to form singlet and triplet states, respectively. This indicates that the S_1_ and T_1_ states will be produced by the ratio of 1: 3 for energy-sufficient ECL systems [26]. Therefore, their maximum ϕ_ECL_ is limited to 25% for fluorescent molecules statistically even if the PLQY is 100%. On the other hand, for phosphorescent molecules, ϕ_ECL_ can reach 100% via efficient intersystem crossing (ISC) from the S_1_ to T_1_ state. Hence, phosphorescent materials tend to show higher ϕ_ECL_ than fluorescent materials. Phosphorescent materials with high PLQY are frequently composed of precious metals such as platinum and iridium. Since ECL using organic materials is cost-effective compared to the case using phosphorescent materials, several studies are focusing on improving the efficiency of ECL using organic molecules.

## Recent progress

### ECL from thermally activated delayed fluorescence molecules

**Fig. 4:**
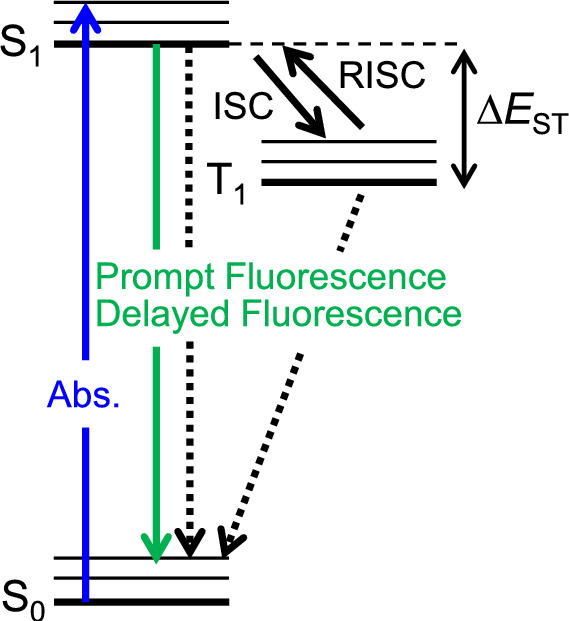
Illustration of photophysical transitions of TADF molecules. Dotted arrows are non-radiative thermal deactivation

For thermally activated delayed fluorescence (TADF) molecules, the reverse intersystem crossing (RISC) from the T_1_ to S_1_ state occurs at room temperature, and delayed fluorescence can be seen (the lifetime of the “prompt” component is in nanoseconds whereas that of delayed fluorescence is normally in microseconds) [27]. The electronic transition upon light absorption is illustrated in Fig. 4. TADF molecules have been developed for organic light-emitting diodes (OLEDs). Because of RISC, the produced T_1_ state by the charge recombination in OLEDs can effectively turn into the S_1_ state. Thus, using TADF molecules for OLEDs, the efficiency of the OLEDs can exceed the limit regulated by the spin statistics, and possibly reach 100% when the PLQY of TADF molecules is almost 100% [28].

It is expected that highly efficient ECL can be realized with organic molecules by the thermal spin-up conversion from the T_1_ state, which is generated by ion annihilation, to the S_1_ state. Several TADF molecules were applied to ECL [29–32], and efficient ECL was achieved by the thermal spin up-conversion. Most TADF molecules are electron donor–acceptor type molecules and form a charge-transfer excited state. Because the charge-transfer excited states have a larger dipole moment, they can be stabilized by the solvent dipoles. Therefore, the photophysical properties such as the rate of the spin up-conversion (the energy gap between the T_1_ and S_1_ (Δ*E*_ST_), as shown in Fig. 4), fluorescence spectrum, and PLQY are affected by the polarity of the solvent [33–35]. For example, for 4CzIPN, which shows efficient spin up-conversion, the value of Δ*E*_ST_ in toluene and MeCN was determined to be 0.14 and 0.098 eV, respectively [33]. This solvent dependence suggests that the S_1_ state is more stabilized by polar solvent molecules than the T_1_ state. Since the ECL properties also depend on the photophysical properties, it is possible to gain a deeper understanding of the ECL properties of donor–acceptor molecules, including TADF molecules, by clarifying the solvent effects.

### Kinetic effects of the homogeneous electron transfer on ECL efficiency

There is also a pathway through which the ground state is formed directly by the homogeneous electron transfer between radical species. This pathway is normally highly exothermic and seems energetically favorable. However, kinetically, the ground state is unlikely to form directly by the electron transfer. For outer-sphere electron transfer reactions, the rate of the electron transfer reaction (*k*_et_) increases as the Gibbs energy difference (Δ*G*^⚬^) between the states of a pair of radical species and a pair of excited and ground states before and after the reaction, respectively, becomes more negative. In the Marcus model, *k*_et_ reaches a maximum value when Δ*G*^⚬^ corresponds to the reorganization energy (λ_o_) of the solvent molecules, and then decreases as Δ*G*^⚬^ becomes more negative (the inverted region). *k*_et_ can be written as [36],

12 $$k_\text{et} = \frac{2\pi }{\hbar }H_\text{if}^{2} \frac{1}{{\sqrt {4\pi \lambda_\text{o} k_{B} T} }}\exp \left( { - \frac{{\left( {\lambda_\text{o} + \Delta G^{\circ } } \right)^{2} }}{{4\lambda_\text{o} k_{B} T}}} \right)$$

where *ħ* = *h*/(2π).* h* and *k*_B_ mean the Plank constant and Boltzmann constant, respectively. *H*_if_ is the mixing energy of the initial and final states, which significantly depends on the degree of overlap of molecular orbitals related to the electron transfer. Therefore, *H*_if_ decreases with the electron transfer distance. Several studies indicate that the medium (solvent) in which the electron transfer occurs significantly affects the decay of *H*_if_ [37]. In direct electron transfer-type bioelectrocatalysis, it was suggested that amino acids having aromatic rings such as tryptophane assist long-distance electron transfer in an enzymatic reaction in an electron transport chain [38].

It is understood that when Δ*G*^⚬^ is extremely negative, the orientation change of the solvent molecules before and after the reaction becomes large, and it takes time to reach solvated states at which electron transfer is favorable. For ECL, *k*_et_ of the formation of emissive excited states is normally large (where − Δ*G*^⚬^ ≈ 0.2–0.3 eV), but that of the ground state is low (where − Δ*G*^⚬^ ≈ 2.5–3 eV, inverted region) [39].

Equation 12 suggests that *k*_et_ for the formation of the excited states can also be lowered as Δ*G*^⚬^ becomes extremely negative. It is expected that ϕ_ECL_ of organic molecules with a large energy separation between the S_1_ and T_1_ states can be enhanced kinetically by suppressing the rate of the T_1_ state formation.

**Fig. 5:**
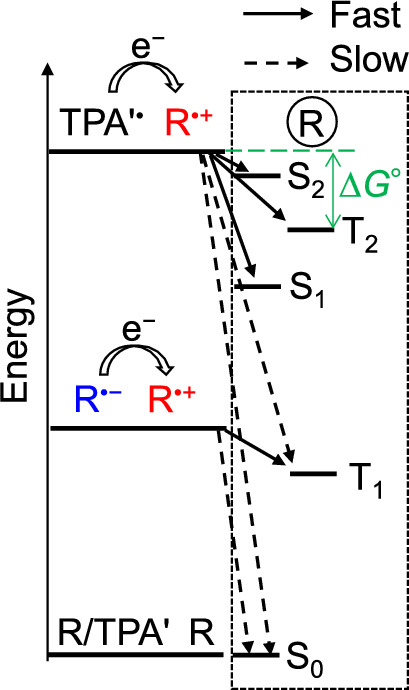
Illustration of rates for the formation of the excited and ground states by the electron transfer reactions. Δ*G*^○^ between a pair of R^•+^ and TPA'^•^ (initial state) and the T_2_ state of R (final state) is shown as an example

The kinetic effect on ϕ_ECL_ was recently found in pyrrolopyrrole aza-BODIPYs (PPABs) that emit near-infrared ECL generated using TPA [40]. For near-infrared PPABs, the energy level of the T_1_ state is low-lying compared to that of a radical pair of R^•+^ and TPA'^•^ (Fig. 5). Some of PPABs showed efficient coreactant ECL. The PLQY and relative ϕ_ECL_ values, energy levels of the S_1_, S_2_, T_1_, and T_2_ states, and redox potentials suggest that the formation of the S_1_, S_2_, and T_2_ states (− Δ*G*^⚬^ ≈ 0.1–0.7 eV) is fast whereas that of the T_1_ state is slow (− Δ*G*^⚬^ ≈ 1.4 eV), which leads to the suppression of the T_1_ state formation and improving ϕ_ECL_ [40]. This kinetic scheme is depicted in Fig. 5.

Because of the unique photophysical properties of BODIPY dyes such as high PLQY and small full width at half maximum of the PL spectrum, BODIPY dyes have been employed for ECL studies [41]. The energy gap between the S_1_ and T_1_ states tends to be large for BODIPY derivatives and their analogues (for some molecules, the energy level of the S_1_ state is about twice that of the T_1_ state) [40, 42]. For BODIPYs, the energy of the pair of R^•−^ and R^•+^ is often lower than the energy level of the S_1_ state (and higher than that of the T_1_ state), so that no ECL or weak ECL is seen by the ion annihilation of R^•−^ and R^•+^ [40, 41]. Although coreactants are normally employed for BODIPYs, mixed annihilation ECL can also be adapted to enhance the ECL intensity.

### Reasons for the discrepancy between ECL and PL spectra

It is essential to confirm whether the PL and ECL spectra agree or disagree. The wavelength sensitivity of the detectors recording the spectra usually varies from instrument to instrument, so calibrated detectors must be used when comparing PL and ECL spectra. When the PL and ECL spectra agree, it can be confirmed that the emitting species is the same in the PL and ECL systems. When they disagree, the following may be the cause.

**Fig. 6:**
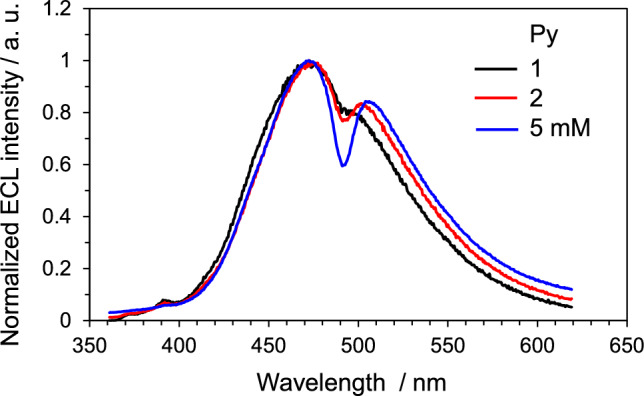
ECL spectra from MeCN solutions of Py at different concentrations. The ECL was generated by stepping the potential from a negative (kept for 0.1 s) to a positive direction. The ECL spectra recorded for 10 ms after the potential step to a positive direction are displayed

**Fig. 7:**
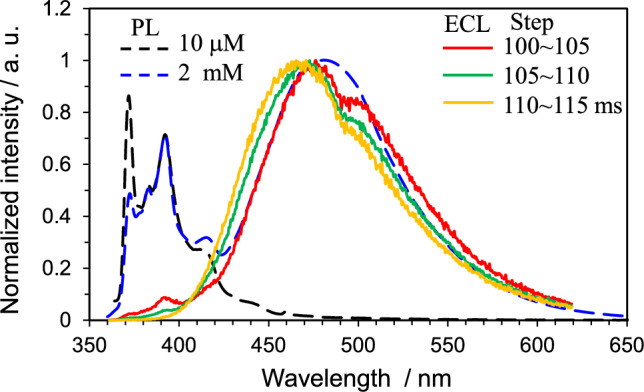
Time-dependent normalized ECL spectra from an MeCN solution of 1 mM Py. The ECL was generated by stepping the potential from a negative (kept for 0.1 s) to a positive direction. The ECL was integrated for 5 ms to record the spectra. ECL occurs after the potential is switched positive (from 100 ms)

(1) Self-absorption. Normally, PL spectra are acquired at about 10 μM to suppress the self-absorption, whereas ECL experiments are performed at a concentration of about 1 mM. When molecules with a small Stokes shift in the absorption and PL spectra are used for the ECL experiments at such normal concentrations, the maximum ECL wavelength will shift to the longer wavelength due to the self-absorption (inner filter effect). In addition, when the absorption spectrum of radical ions produced in the electrode reaction overlaps with the ECL spectrum, the resultant ECL spectrum will be deformed. For example, the light absorption by the radical anion of pyrene (Py) distorts the ECL spectrum [43, 44]. Figure 6 displays ECL spectra from an MeCN solution of Py. The spectra recessed around 490 nm, and the recession was enhanced as the concentration of Py increased, which was caused by the ECL absorption by the radical anion of Py (Py^•−^) showing a light absorption maximum of 490 nm. Overall, when the shifts are only due to the inner-filter effect, it can be concluded that the emitting species is the same for PL and ECL.

(2) ECL from decomposition products or polymerized compounds. When the electrochemically generated radical ions are unstable, the subsequent chemical reactions produce decomposition products, polymers, etc. When the ECL from these products is appreciable, the observed ECL spectrum consists of the ECL from the parent molecule and the products. As the electrode reaction proceeds, the amount of these products increases, so the resultant ECL spectrum also changes over time [45]. Compared to the ECL spectrum of monomers, that of polymers are generally red-shifted and broader. Figure 7 shows the PL spectra of Py at 10 μM and 2 mM and the time-dependent ECL spectra from 1 mM Py dissolved in MeCN. The broad PL component with the maximum wavelength at 490 nm confirmed at 2 mM was the excimer emission and structured PL around 360–450 nm was the monomer emission. On the ECL spectra, the maximum wavelength around 490 nm shifted to a shorter wavelength over time, which indicates the emissive species changes over time (here, the blue-shifted ECL compared to the excimer emission is caused by the oligomers of Py).

(3) Excimer and exciplex emission. For ECL, since there is a ground state molecule near R* just after the electron transfer reaction, excimers [46–48] and exciplexes [49–51] are easily formed. In the case of the excimer formation, R of isolated pairs (R* and R, which are produced by the electron transfer) and R near the isolated pairs are responsible for the excimer formation. R* is generated in the diffusion layer near the electrode, and the concentration of R in the diffusion layer changes with time. Therefore, the monomer and excimer components of the ECL change with time (see following).

### Monomer and excimer emission in ECL

**Fig. 8:**
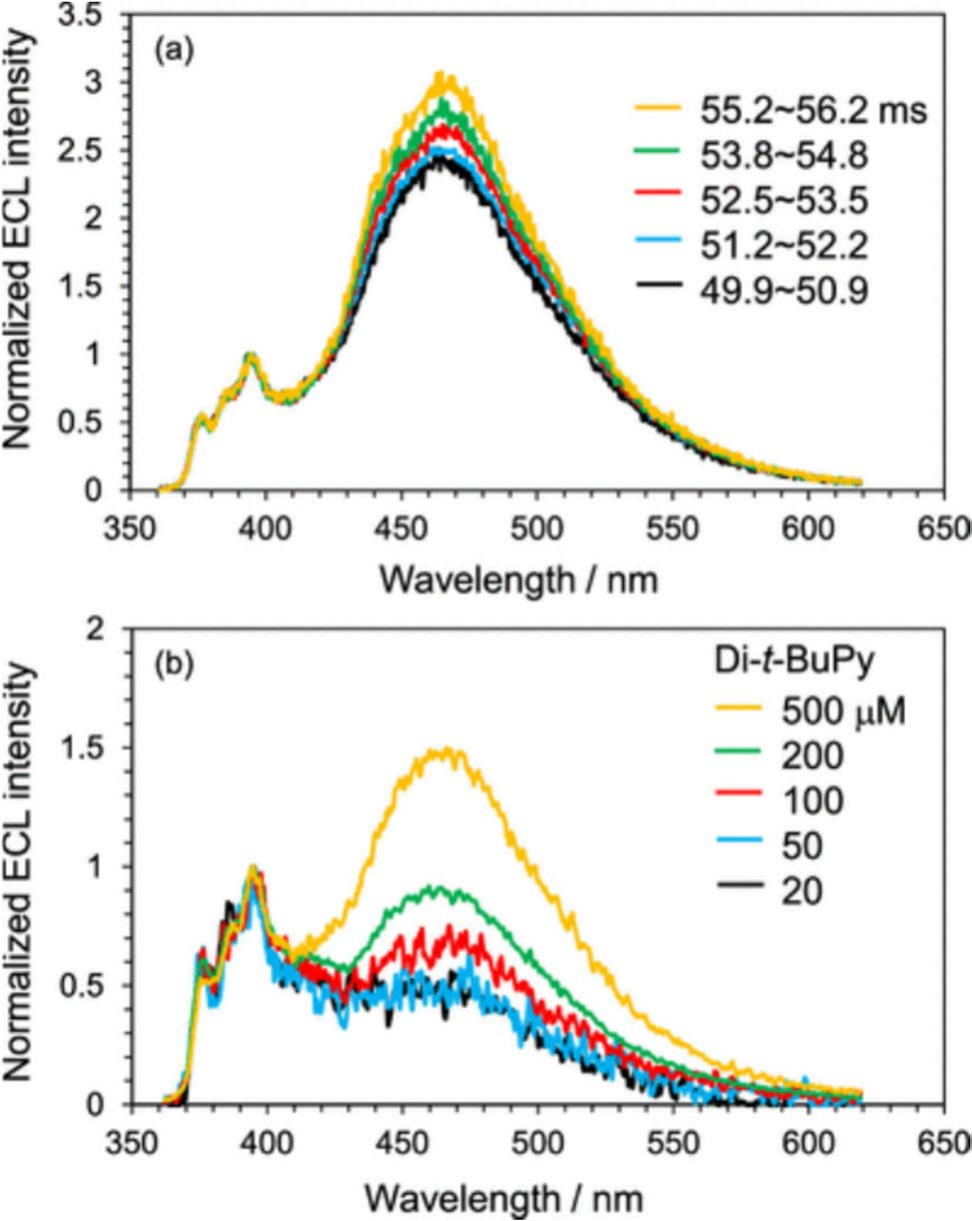
(**a**) Normalized time-dependent and (**b**) concentration-dependent ECL spectra of Di-*t*-BuPy just after the potential step. The ECL was generated by stepping the potential from a negative (kept for 50 ms) to a positive direction. The ECL was integrated for 1 ms to record the spectra. ECL occurs after the potential is switched positive (from 50 ms). Reprinted (adapted) with permission from Ref. [44]. Copyright 2023 American Chemical Society

Recently, the time dependence and solution concentration dependence of the ECL spectra for Py and 2,7-di-*tert*-butylpyrene (Di-*t*-BuPy) have been clarified [44]. Figure 8 shows normalized time-dependent and concentration-dependent ECL spectra of Di-*t*-BuPy. The broad and structured components around 470 and 390 nm are the excimer and monomer emission, respectively. When the concentration of Di-*t*-BuPy was relatively high (1 mM, Fig. 8a), the excimer component against the monomer one increased with time because the concentration of the neutral state of Di-*t*-BuPy, which was significantly lowered just after the potential step, increased with time and led to the enhancement of the excimer emission.

When the initial concentration of Di-*t*-BuPy was decreased, the relative excimer intensity on the ECL spectra just after the potential step was lowered, and the shape of the ECL spectra at 20 and 50 μM agreed well (Fig. 8b). This means that isolated pairs of R^•−^ and R^•+^, which resulted in the formation of pairs of R* and R, are responsible for the shape of the ECL spectra (the ratio of the monomer and excimer emission), and neutral R near the isolated pairs do not participate in the ECL emission.

Several photophysical measurements can reveal ϕ_PL_ of the monomer (ϕ_PL_M_) and excimer emission (ϕ_PL_Ex_). For Di-*t*-BuPy, ϕ_PL_M_ and ϕ_PL_Ex_ were determined to be 0.50 and 0.24, respectively. The values were also estimated for Py, as 0.64 and 0.53, respectively. The values give the ratio of the monomer excited ([M*]) and excimer states ([Ex]) from 1/ϕ_PL_Ex_ and 1/ϕ_PL_M_, and found to be [M*]: [Ex] = 0.53: 0.47 for Di-*t*-BuPy and 0.42: 0.58 for Py. Several factors such as the lifetime of the monomer excited state, diffusion, solvation, stabilization energy for forming the excimer state, and initial separation distance of R* and R (which coincides with the electron transfer distance) may affect the ratio.

When the formation of the excimer is assumed to be limited by diffusion, the time-dependent survival probability (*q*(*t*)) of R* in the isolated pairs can be written as [52, 53],

13 $$q\left(t\right)=1-\frac{{d}_{\text{react}}}{{d}_{\text{initial}}}\text{erfc}\left(\frac{{d}_{\text{initial}}-{d}_{\text{react}}}{\sqrt{4{D}^{\prime}t}}\right)$$

where erfc means the complementary error function and *d*_initial_ and *d*_react_ indicate the average initial separation and reaction distances of R* and R pairs, respectively. *D*′ represents the relative diffusion coefficient of the pair. Here, the function, $\text{erfc}\left(\frac{{d}_{\text{initial}}-{d}_{\text{react}}}{\sqrt{4{D}^{\prime}t}}\right)$, converges to 1 in a few ns. The lifetime of the excited state of Di-*t*-BuPy and Py was about 250 and 290 ns, respectively, which are sufficiently long to regard as $q\left(t\right)=1-{d}_{\text{react}}/{d}_{\text{initial}}$. By employing the intermolecular distance for the excimer of Di-*t*-BuPy and Py of 3.51 and 3.37 Å, respectively, as *d*_react_, which were determined from time-dependent DFT calculation, *d*_initial_ was determined to 7.1 and 6.1 Å, respectively. Because of the interactive force between R* and R, which depends on the intermolecular distance and orientation of the molecules, the actual electron transfer distance may be larger. The author expects that the dynamics of the electron transfer accompanied by diffusion can be elucidated in more detail by combining the time-dependent ECL measurements with theoretical calculations such as quantum mechanics/molecular mechanics.

### ECL of Eu and Tb complexes

Metal complexes with lanthanides such as Eu(III) and Tb(III) as metal centers have interesting photophysical properties such as very sharp PL spectra. Furthermore, the shape of the PL spectra is hardly affected by the kinds of ligands [54, 55]. Eu(III) and Tb(III) complexes have high color purity in pink and green emission, respectively, and have attracted attention for light-emitting devices such as OLEDs [56].

The unique properties of lanthanides are also of great interest in the field of ECL, however, systematic studies on the ECL of lanthanides are limited [57, 58]. We used several Eu(III) and Tb(III) complexes composed of β-diketonates and N-ligands such as 1,10-phenanthroline for ECL and discussed the ECL mechanism depending on the ligands [59, 60].

The oxidized and reduced forms of the complexes used were unstable, and very weak annihilation ECL was observed when a square wave voltage was applied. These obtained ECL spectra were very sharp, and it was confirmed that the emission was due to transitions between 4f orbitals, as in the case of PL. The electronic transition between 4f orbitals is further expressed by a term (^2*S*+1^L_*J*_) using the spin quantum number *S*, total orbital angular momentum L, and total angular momentum *J*. Only the electronic transition with the highest emission intensity (^5^D_0_ → ^7^F_2_ for Eu complexes, corresponding to 612 nm, and ^5^D_4_ → ^7^F_5_ for Tb complexes, corresponding to 545 nm) was confirmed for the annihilation ECL.

**Fig. 9:**
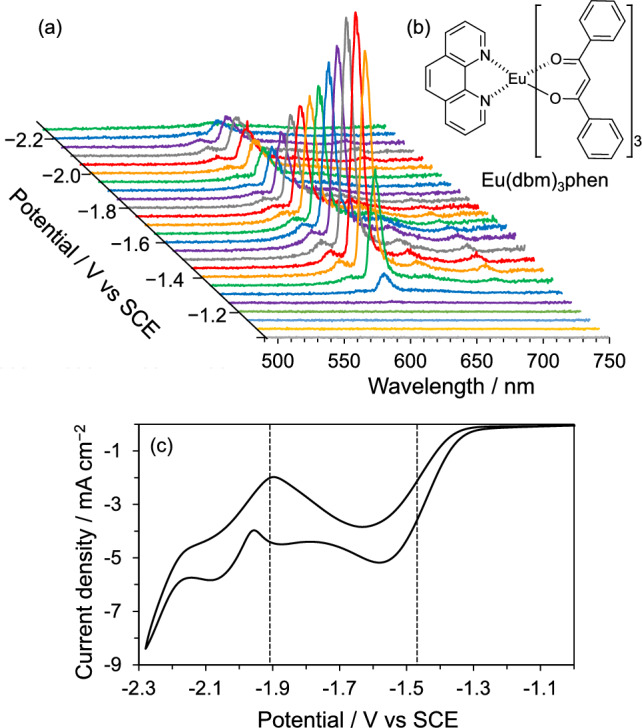
(**a**) Potential-dependent ECL spectrum of 2 mM Eu(dbm)_3_phen with 20 mM S_2_O_8_^2−^, (**b**) molecular structure of Eu(dbm)_3_phen, and (**c**) the current–potential curve. The broken lines indicate the peak potentials estimated from the coreactant-free system. Scan rate: 50 mV s^−1^. Reproduced from Ref. [59] by permission of John Wiley & Sons Ltd

When S_2_O_8_^2−^ was used as a coreactant, the ECL intensity increased significantly, and the transitions to other electronic states, which were confirmed in the PL spectra such as the electronic transition to ^7^F_1_, were also observed in ECL. Figure 9 shows potential-dependent ECL spectra and its current–voltage curve of 20 mM S_2_O_8_^2−^ with 2 mM Eu(dbm)_3_phen (the molecular structure is also shown in Fig. 9) in MeCN. The vertical broken lines in Fig. 9c indicate the reduction peaks observed in the system of 2 mM Eu(dbm)_3_phen, where the first and second reductions are attributable to the metal center and the N-ligand, respectively. The reduction current of S_2_O_8_^2−^ flowed at around − 1.5 V vs. SCE. The ECL intensity at λ_ECL_= 612 nm reached the maximum around the potential. SO_4_^•−^, which is the product of the electrode reduction of S_2_O_8_^2−^, can remove an electron located on the dbm ligands, as judged from the results of the DFT calculations. Then the excited state of Eu(dbm)_3_phen will be formed by intramolecular electron transfer from the metal center to the ligands, which results in ECL by the 4f transition.

For the Tb(III) complexes employed, the annihilation and coreactant ECL intensity were much weaker than those of Eu(dbm)_3_phen, respectively, although the PLQY value of the Tb(III) complexes (4–12%) are comparable to that of Eu(dbm)_3_phen (6%). The metal center Tb(III) is less oxidized and reduced than the coordinated ligands. Therefore, in the potential scan, the reduction originating from the ligand occurs first, and SO_4_^•−^ can remove an electron from the reduced ligand, and a locally excited state on the ligand will be formed. After that, through ISC and energy transfer, ECL originating from the 4f transition can be observed.

## Mixed ECL systems

For mixed annihilation ECL systems (Eqs. 5–7), one can select A and D which produce stable radical ions from many candidates with a variety of redox potentials. Unlike the coreactants, which irreversibly produce products, D and A generated after the electron transfer reaction (Eqs. 7 and 8) can be electrochemically re-reduced and re-oxidized. Mixed annihilation ECL systems have recently attracted attention. Francis and coworkers have studied mixed ECL systems. They changed the combination of radical ion pairs of the metal complexes by selecting the applied potential and systematically investigated the effect of the combination on the ECL properties [61–65].

When the redox potential difference of *E*_1/2_ between R/R^•−^ and D/D^•+^ or R/R^•+^ and A/A^•−^ is sufficient to form the S_1_ state (for fluorescent molecules) or T_1_ state (for phosphorescent molecules) of R, ECL from R can be seen. The relationship between the redox potential gap on ϕ_ECL_ was clarified using Ir(ppy)_3_ and several A by Kapturkiewicz et al. [66]. They annihilated A^•−^ with the oxidized form of Ir(ppy)_3_ (Ir(ppy)_3_^•+^, *E*_1/2_(Ir(ppy)_3_/Ir(ppy)_3_^•+^) = 0.23 V vs Fc/Fc^+^), where A^•−^ can be produced more easily than the reduced form of Ir(ppy)_3_ (Ir(ppy)_3_^•−^, *E*_1/2_(Ir(ppy)_3_/Ir(ppy)_3_^•−^) =  − 2.66 V vs Fc/Fc^+^). They found that ϕ_ECL_ of Ir(ppy)_3_ increased with the potential gap between Ir(ppy)_3_^•+^ and A^•−^ increased. Among A they used, 2-cyanofluorene (*E*_1/2_ ≈ − 2.55 V vs Fc/Fc^+^) showed the highest ϕ_ECL_.

**Fig. 10:**
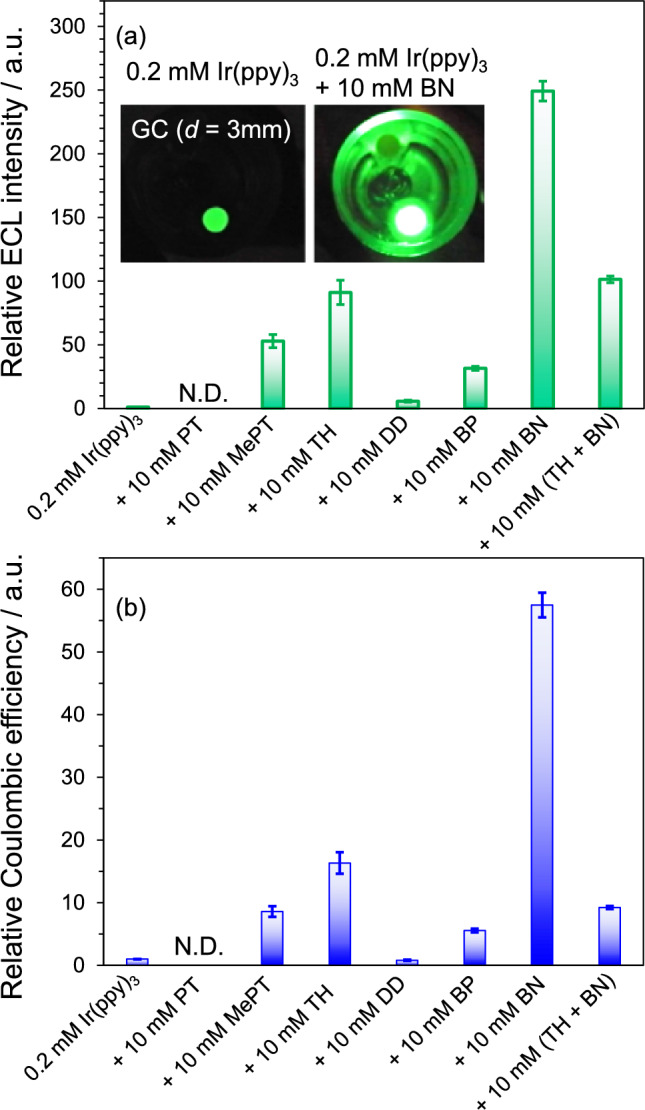
(**a**) Relative ECL intensity and (**b**) relative Coulombic efficiency (total ECL intensity divided by total charge) of mixed systems against the single system of 0.2 mM Ir(ppy)_3_ dissolved in MeCN. Inset in panel (**a**): photographs of ECL of 0.2 mM Ir(ppy)_3_ (left) and 0.2 mM Ir(ppy)_3_ with 10 mM BN (right). *N.D.* not detected. Reprinted from Ref. [67], Copyright 2024, with permission from Elsevier

On the other hand, we used several D, phenothiazine (PT, *E*_1/2_(PT/PT^•+^) = 0.23 V vs Fc/Fc^+^), 10-methylphenothiazine (MePT, *E*_1/2_(MePT/MePT^•+^) = 0.34 V vs Fc/Fc^+^), thianthrene (TH, *E*_1/2_(TH/TH^•+^) = 0.85 V vs Fc/Fc^+^), and dibenzo-1,4-dioxin (DD, *E*_1/2_(DD/DD^•+^) = 1.02 V vs Fc/Fc^+^), and two different A (benzophenone (BP, *E*_1/2_(BP/BP^•−^) =  − 2.21 V vs Fc/Fc^+^) and benzonitrile (BN, *E*_1/2_(BN/BN^•−^) =  − 2.78 V vs Fc/Fc^+^)[67]. It is noted that BN can be reduced at a more extreme potential than Ir(ppy)_3_. Figure 10 (a) and (b) show the relative ECL intensity and efficiency for the binary systems of 0.2 mM Ir(ppy)_3_ with 10 mM A or D and the ternary system with 10 mM TH and BN against the single system of 0.2 mM Ir(ppy)_3_ dissolved in MeCN. In both the case of using A and D, the more extreme redox potentials are favorable to enhance the ECL intensity. In the case of using DD, DD^•+^, which is a strong electron-accepter, can produce unstable Ir(ppy)_3_^2+^ and lead to weaker ECL. BN is the best additive among A and D employed. Because the ECL intensity for the binary system using TH was largely enhanced among the binary systems using D, the ternary system using BN and TH was investigated. The ECL intensity was smaller than that of the binary system using BN and comparable to that using TH. The electron transfer between BN^•−^ and TH^•+^ is a drawback for enhancing the ECL intensity.

**Fig. 11:**
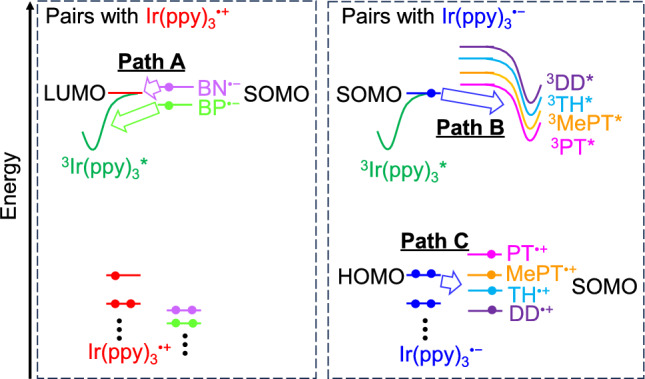
Possible electron-transfer pathways in the binary system of Ir(ppy)_3_ and D or A. SOMO: singly occupied molecular orbital. Reprinted from Ref. [67], Copyright 2024, with permission from Elsevier

Three possible pathways were discussed based on the electron-donating and -accepting characteristics of A^•−^ and D^•+^, the energy level of the T_1_ state of A, D, and Ir(ppy)_3_, and the redox potentials (Fig. 11). Because BN^•−^ work as an electron donor, the excited state will be formed in Ir(ppy)_3_ by the electron transfer (Path A), and the energy level of the T_1_ state of Ir(ppy)_3_ is lower than that of BN, the triplet energy transfer from Ir(ppy)_3_ to BN cannot occur. Furthermore, the electron transfer can occur between BN^•−^ and Ir(ppy)_3_ due to the reduction potentials, and a large amount of Ir(ppy)_3_^•−^ will be generated. For the systems using D, via Path B and Path C, the triplet excited state of D (^3^DD*, ^3^TH*, ^3^MePT*, and ^3^PT*), and triplet excited state of Ir(ppy)_3_ (^3^Ir(ppy)_3_*) can be formed. The higher ECL intensity for the system using BN is likely to be attributable to the absence of forming the triplet state of BN.

## Conclusions

The ECL properties of fluorescent and phosphorescent materials have been reported, and those of newly synthesized PL materials are still being reported by many groups. ECL involves several processes such as electrode reactions, diffusion of electrochemically generated radical ions, the formation of excited states by the electron transfer between the radical ion species, and the light-emitting electronic transitions, and these processes must be elucidated to understand the ECL phenomena deeply.

In this mini-review, the author introduced the ECL properties of two groups of molecules with unique electronic structures. One is TADF molecules, and the other is near-infrared light-emitting PPAB molecules. For TADF molecules, RISC from the T_1_ to S_1_ state occurs efficiently, and the T_1_ state generated by the electron transfer also undergoes spin up-conversion to the S_1_ state, realizing highly efficient ECL. For near-infrared light-emitting PPAB molecules, the energy gap between the S_1_ and T_1_ is significantly large. In this case, for the coreactant ECL system with TPA, the energy of the radical pair generated by the electrode reaction is much greater than the T_1_ level of one of the PPAB molecules, and the rate for the T_1_ formation by electron transfer significantly slows down, thereby kinetically increasing the ECL efficiency. Recently, ECL that exhibits circular polarization was reported [68], and ECL of materials with unique optical properties and electronic states is very interesting. The author expects that various PL materials will be developed in the future and that unique ECL properties will be reported.

Time-resolved ECL measurements are a powerful method for revealing ECL dynamics. The shape of ECL spectra is sometimes influenced by electrode reactions, inner-filter effect, and excimer formation. Here, time-dependent ECL spectra from a Py solution were introduced. Because the degree of the electrochemical polymerization of Py (broad spectra arising from oligomers) and the amount of Py^•−^ (inner-filter effect deforming the ECL spectra) and Py near isolated pairs of Py^•−^ and Py^•+^ (enhancing the excimer emission), respectively, changes over time, the ECL spectra were time-dependent. It was possible to estimate the ratio of the monomer and excimer emission generated from isolated pairs (Py^•−^ and Py^•+^, and Di-*t*-BuPy^•−^ and Di-*t*-BuPy^•+^). In this way, it was possible to elucidate the ECL mechanism in more detail.

For the mixed systems using R and A or D, the energy level of the excited states, redox potentials, and electron donating and accepting characteristics of A^•−^ and D^•+^ should be considered. When the mixed system is energetically sufficient to produce R*, combinations of R and A or D, where the triplet excited states will not be produced, are favorable to enhance the ECL intensity further. In the case of using Ir(ppy)_3_, BN is a good additive to enhance the ECL intensity. Overall, the ECL intensity (efficiency) and spectrum are strongly influenced by the mechanisms behind them. Elucidating these will lead to a deeper understanding of the ECL phenomena.

## Data Availability

The data used in the current study are available from the corresponding author upon reasonable request.
